# Nonselective versus Selective Angioembolization for Trauma Patients with Pelvic Injuries Accompanied by Hemorrhage: A Meta-Analysis

**DOI:** 10.3390/medicina59081492

**Published:** 2023-08-19

**Authors:** Hyunseok Jang, Soon Tak Jeong, Yun Chul Park, Wu Seong Kang

**Affiliations:** 1 Division of Trauma, Department of Surgery, Chonnam National University Medical School and Hospital, Chonnam National University, Gwangju 61469, Republic of Korea; bluetholic@naver.com (H.J.); kontikithor@gmail.com (Y.C.P.); 2 Department of Physical Medicine and Rehabilitation, Ansanhyo Hospital, Ansan-si 15457, Republic of Korea; loansanchief@gmail.com; 3 Department of Trauma Surgery, Jeju Regional Trauma Center, Cheju Halla General Hospital, Jeju 63127, Republic of Korea

**Keywords:** pelvic injury, hemorrhage, angioembolization, meta-analysis, systematic review

## Abstract

*Background and Objectives*: Angioembolization has emerged as an effective therapeutic approach for pelvic hemorrhages; however, its exact effect size concerning the level of embolized artery remains uncertain. Therefore, we conducted this systematic review and meta-analysis to investigate the effect size of embolization-related pelvic complications after nonselective angioembolization compared to that after selective angioembolization in patients with pelvic injury accompanying hemorrhage. *Materials and Methods*: Relevant articles were collected by searching the PubMed, EMBASE, and Cochrane databases until 24 June 2023. Meta-analyses were conducted using odds ratios (ORs) for binary outcomes. Quality assessment was conducted using the risk of bias tool in non-randomized studies of interventions. *Results*: Five studies examining 357 patients were included in the meta-analysis. Embolization-related pelvic complications did not significantly differ between patients with nonselective and selective angioembolization (OR 1.581, 95% confidence interval [CI] 0.592 to 4.225, I^2^ = 0%). However, in-hospital mortality was more likely to be higher in the nonselective group (OR 2.232, 95% CI 1.014 to 4.913, I^2^ = 0%) than in the selective group. In the quality assessment, two studies were found to have a moderate risk of bias, whereas two studies exhibited a serious risk of bias. *Conclusions*: Despite the favorable outcomes observed with nonselective angioembolization concerning embolization-related pelvic complications, determining the exact effect sizes was limited owing to the significant risk of bias and heterogeneity. Nonetheless, the low incidence of ischemic pelvic complications appears to be a promising result.

## 1. Introduction

Pelvic injuries accompanied by severe hemorrhage pose significant challenges to trauma management and are associated with high morbidity and mortality rates. Achieving hemostasis in these cases is crucial in preventing life-threatening complications and improving patient outcomes [1,2]. Transarterial angioembolization (TAE) has emerged as an effective therapeutic option for the control of bleeding in patients with pelvic injuries [3]. Various modalities, such as angioembolization, preperitoneal pelvic packing (PPP), and resuscitative endovascular aortic balloon occlusion (REBOA) zone 3, have been introduced and adopted to control pelvic hemorrhages [1,4,5,6]. However, the exact effect sizes of these modalities remain unclear. In a recent retrospective cohort study in the US using the 2017 American College of Surgeons Trauma Quality Improvement Program database, only pelvic angioembolization was associated with mortality reduction, whereas PPP or REBOA zone 3 was not significantly associated with mortality in the multivariable regression analysis [6]. Pelvic angioembolization is apparently the most crucial modality in modern trauma care [6].

TAE involves the selective catheterization of the pelvic arteries, followed by the injection of embolic agents to occlude the bleeding vessels [3,7]. This minimally invasive procedure offers several advantages, including the ability to target specific bleeding sources, preserve the surrounding tissue, and avoid the need for more invasive surgical interventions. Despite the increasing use of TAE, comprehensive research is needed to explore its clinical effectiveness, patient selection criteria, procedural considerations, and long-term outcomes. In cases of unstable pelvic injury accompanied by severe hemorrhage, clinicians may opt for proximal nonselective angioembolization as the preferred approach for expeditious bleeding control [1]. This preference stems from the relatively lower level of technical complexity and expeditiousness associated with proximal nonselective angioembolization compared to that required for selective angioembolization. The concept of “damage control” may be also useful in surgery. However, the evidence in support of nonselective angioembolization is limited. 

We conducted this systematic review and meta-analysis to investigate the effect size of embolization-related pelvic complications after nonselective angioembolization compared to that after selective angioembolization in patients with pelvic injury and accompanying hemorrhage. Through this systematic review, we aimed to provide valuable insights into the efficacy, safety, and clinical outcomes of this procedure, which will aid in guiding clinical decision making and optimizing patient care in this challenging patient population.

## 2. Materials and Methods

### 2.1. Published Study Search and Selection Criteria

This study was conducted in accordance with the Preferred Reporting Items for Systematic Reviews and Meta-Analyses guidelines [8]. The study protocol was prospectively registered in PROSPERO (CRD42022322786; https://www.crd.york.ac.uk/prospero accessed on 3 May 2022). Relevant articles were obtained through comprehensive searches of the MEDLINE PubMed, EMBASE, and Cochrane databases for the period up to 24 June 2023. These databases were searched using the following keywords: (“pelvic bones” OR pelvic OR pelvis) AND (fracture* OR injur* OR trauma*) AND (angiograph* OR angioemboli* OR “angio emboli*” OR “angio-emboli*” OR angiothera* OR “transarterial embo*” OR “transcatheter arterial embo*” OR emboli*). Furthermore, an additional search was performed by manually scrutinizing the reference lists of relevant articles. The titles and abstracts of all the retrieved articles were meticulously screened to determine their eligibility for inclusion. Review articles and meta-analyses were also assessed to identify supplementary studies that met the eligibility criteria. Subsequently, the search results were thoroughly reviewed, and articles investigating angioembolization in patients with pelvic injury and concurrent hemorrhage were included.

The primary outcomes were embolization-related pelvic complications following pelvic angioembolization. The secondary outcome was in-hospital mortality after pelvic angioembolization. The inclusion criteria for this review were as follows: (i) trauma patients diagnosed with pelvic injury and concurrent hemorrhage; (ii) patients who underwent angioembolization as a treatment modality for pelvic hemorrhage; (iii) comparison between nonselective angioembolization performed in the main trunk of the internal iliac artery (bilateral or unilateral) and selective angioembolization; (iv) availability of pertinent outcomes, such as embolization-related complications or mortality, within the reported data; and (v) provision of odds ratios (ORs), means with standard deviations, or provision of data enabling their calculation. Studies examining diseases other than the specified conditions, nonoriginal research articles, and publications in languages other than English were excluded from the analysis.

### 2.2. Data Extraction

Data extraction was performed by two investigators encompassing all eligible studies. The following key information was extracted from each study: first author’s name, year of publication, study location, study design, study period, number of patients included in the analysis, patient age, injury severity score (ISS), patient vital signs, anatomical location of angioembolization (nonselective or selective), type of embolic agent used, occurrence of embolization-related complications, and mortality rate. Specifically, nonselective angioembolization for pelvic hemorrhage was defined as embolization of the main stem of the internal iliac artery. Nonselective angioembolization encompassed cases in which embolization targeted either the main stem of the bilateral internal iliac arteries or that of the unilateral internal iliac artery. Selective angioembolization was defined as a more selective embolization when the embolization procedure focused on a distal location away from the main stem. Embolization-related pelvic complications were defined as potential adverse outcomes associated with embolization, including wound infection, gluteal or skin necrosis, pelvic infection, fracture nonunion, and osteomyelitis.

### 2.3. Quality Assessment

To evaluate the risk of bias in observational studies, we employed a tool previously used to assess the risk of bias in nonrandomized studies of interventions (ROBINS-I) [9]. All studies were independently reviewed by two investigators. Any disagreements concerning study selection and data extraction were resolved through consensus.

### 2.4. Statistical Analysis

The statistical analyses were carried out utilizing the “meta” package in R, version 4.1.1. (R foundation, Vienna, Austria). Visualizations depicting the risk of bias were generated using the “robvis” R package. Meta-analyses were performed using ORs for binary outcomes and standardized mean differences (SMDs) for continuous outcome measures. Confidence intervals (CIs) were calculated using the exact confidence limits for binomial proportions. Pooling of ORs and SMDs was accomplished using the inverse variance method for the meta-analysis of outcomes [10]. The presence of heterogeneity was assessed visually through forest plots and quantitatively through I^2^ statistics and Cochran’s Q test (*p* < 0.10 was considered statistically significant) [11]. Heterogeneity levels of I^2^ > 25%, >50%, and >75% were considered indicative of low, moderate, and high heterogeneity, respectively [10]. Owing to the limited number of eligible studies (<20), the assessment of publication bias using statistical methods, such as funnel plots and the Egger regression test, was not feasible [12]. To ensure the robustness of our findings, a sensitivity analysis was performed by systematically excluding each study from the analysis [10].

## 3. Results

### 3.1. Selection and Characteristics

**Figure 1:**
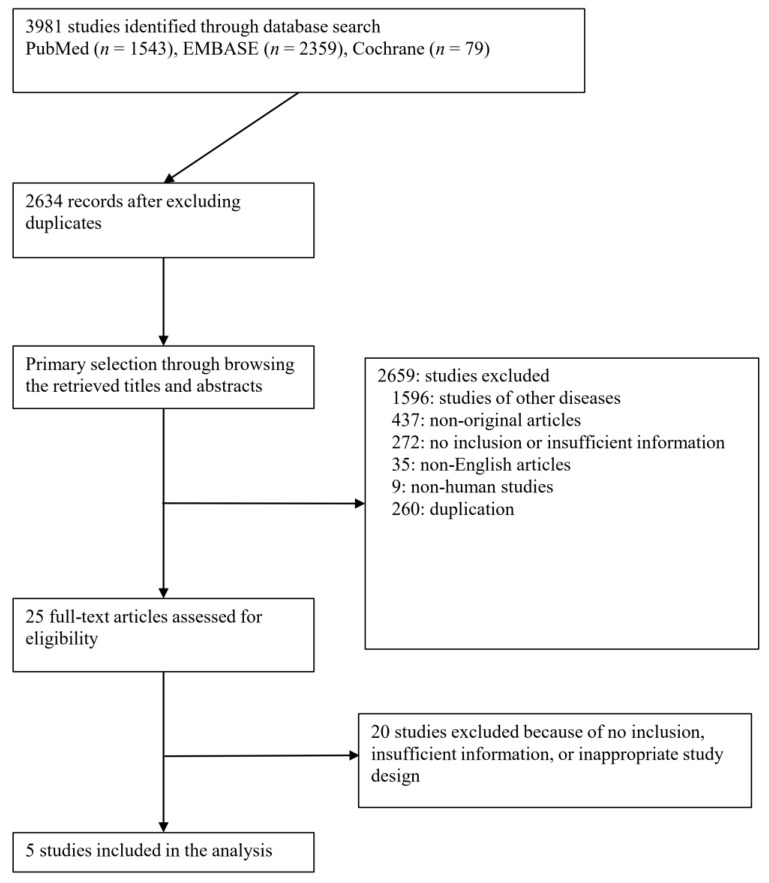
Flowchart summarizing literature and study selection.

A thorough database search identified 3981 studies. Of these, 2659 were deemed ineligible and subsequently excluded from the analysis. The reasons for exclusion were as follows: 1596 studies focused on diseases other than the subject of interest, 437 studies were classified as non-original, 272 studies lacked the necessary inclusion criteria or provided insufficient information, 35 studies were published in non-English languages, and 260 studies were duplicates. Finally, five studies [13,14,15,16,17] involving a collective sample size of 357 patients met the predetermined eligibility criteria and were included in the present meta-analysis (Figure 1). 

### 3.2. Included and Excluded Studies

Detailed information on the eligible studies is summarized in Table 1. All studies were observational, and there were no randomized studies. One study [16] was conducted at three level 1 trauma centers, and the others were single-center studies. During our systematic review, we identified several studies that investigated the use of nonselective angioembolization for pelvic hemorrhage and its associated complications. However, these studies lacked a comparative design and did not provide information regarding the effect size of nonselective angioembolization compared to selective angioembolization. Therefore, we excluded these studies [18,19,20,21,22,23,24]. Detailed information on the excluded studies is summarized in Table 2. 

### 3.3. Quality Assessment

**Figure 2:**
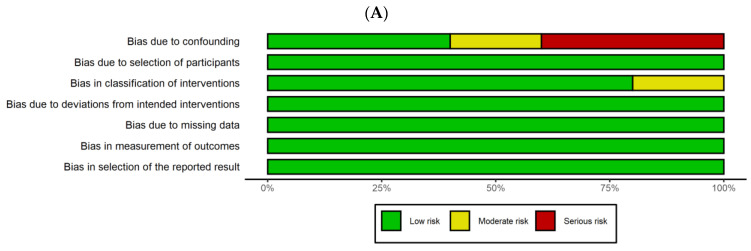

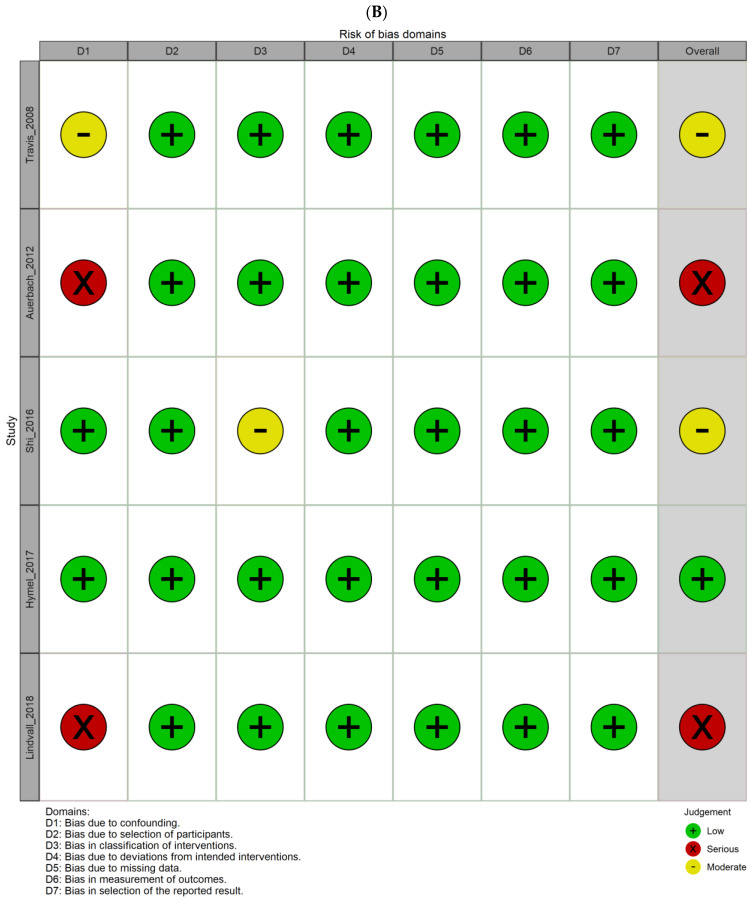
Risk of bias and applicability concerns graph (**A**) and summary (**B**). Review authors’ judgements about each domain presented as percentages across included studies.

Travis et al. [13] did not include important preintervention variables, such as age, injury severity, or vital signs, for patients undergoing nonselective and selective pelvic embolization, which could potentially act as confounding factors. Therefore, we concluded that this study had a moderate risk of bias due to the confounding factors. Auerbach et al. [14] reported that only two patients underwent nonselective pelvic angioembolization, whereas Lindvall et al. [17] reported only two patients who underwent selective pelvic angioembolization. Indeed, these two studies did not report the hemodynamic status. As a result, we determined that these two studies had a serious risk of bias owing to confounding factors. Furthermore, a study conducted by Shi [15] in 2016 classified interventions into three categories, nonselective, divisional, and superselective groups, unlike other eligible studies. Therefore, we conclude that this study carried a moderate risk of bias in the classification of interventions. In summary, two studies [13,15] had a moderate risk of bias, whereas two studies [14,17] exhibited a serious risk of bias. Only one study [16] demonstrated a low risk of bias (Figure 2).

### 3.4. Comparison between Nonselective and Selective Angioembolization for Pelvic Injury with Hemorrhage

**Figure 3:**
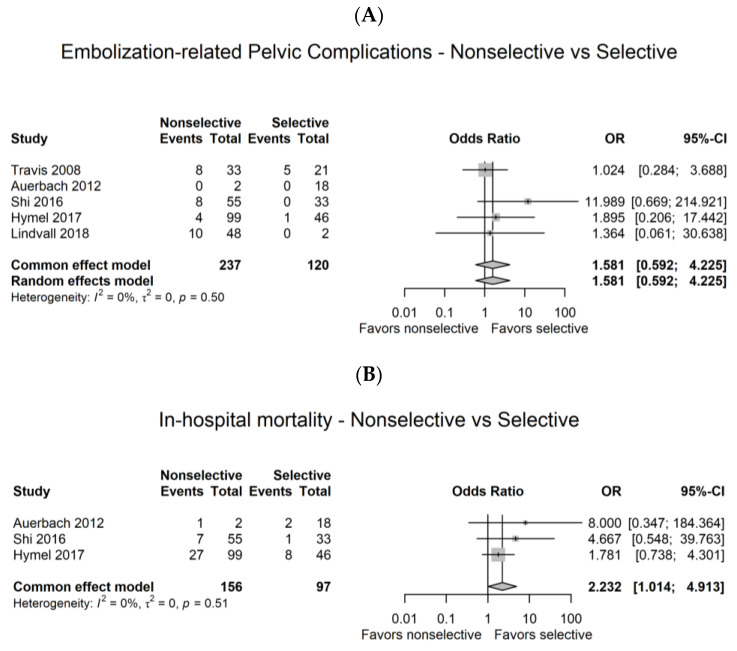
Comparison of nonselective and selective angioembolization. (**A**) Embolization-related pelvic complications [13,14,15,16,17], (**B**) in-hospital mortality. [14,15,16] CI, confidence interval; OR, odds ratio.

Meta-analysis showed that embolization-related pelvic complications did not significantly differ between patients who underwent nonselective and selective angioembolization (OR 1.581, 95% CI 0.592 to 4.225, I^2^ = 0%, Figure 3A). However, in-hospital mortality was more likely to be higher in the nonselective group (OR 2.232, 95% CI 1.014 to 4.913, I^2^ = 0%, Figure 3B).

### 3.5. Sensitivity Analysis

**Figure 4:**
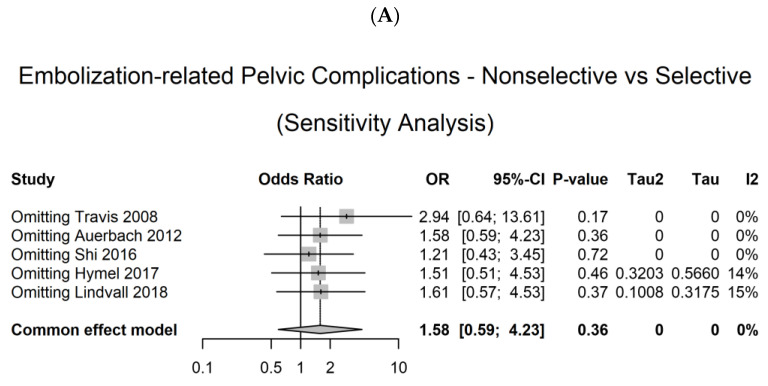

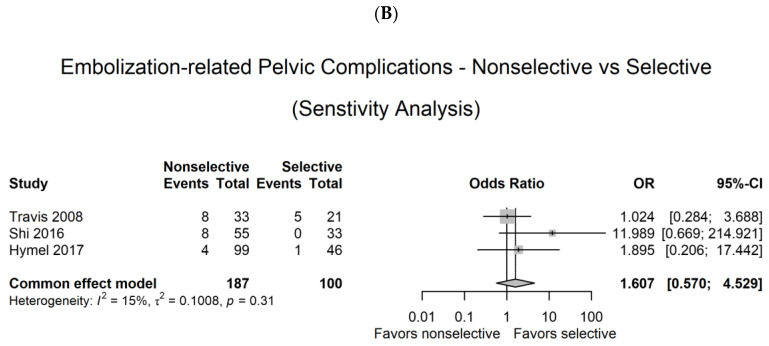
Sensitivity analysis. (**A**) Omitting individual studies [13,14,15,16,17], (**B**) excluding two studies [13,15,16] that exhibited a serious risk of bias. CI, confidence interval; OR, odds ratio.

A sensitivity analysis was performed by systematically removing individual studies and assessing their impact on the overall findings in terms of pelvic complications. Sensitivity analysis based on the quality assessment revealed that the exclusion of any single study did not have a significant influence on the results (Figure 4A). Furthermore, we excluded two studies that exhibited a serious risk of bias. Interestingly, the exclusion of these studies yielded similar results, indicating that their inclusion did not significantly affect the overall outcomes (Figure 4B). Overall, our sensitivity analysis demonstrated the robustness of the findings, as the removal of individual studies and the exclusion of studies with a serious risk of bias did not significantly alter the results. For mortality, we did not perform a sensitivity analysis because of the small sample size.

## 4. Discussion

In our meta-analysis, nonselective pelvic angioembolization for bleeding control showed comparable rates of embolization-related complications when compared to selective angioembolization. Nevertheless, the patients who underwent selective angioembolization exhibited favorable in-hospital mortality outcomes. To the best of our knowledge, this is the first systematic review and meta-analysis to address this issue. However, clinicians should exercise caution when considering this finding because of the substantial risk of bias and heterogeneity identified in the eligible studies. Furthermore, well-designed prospective studies are warranted to address these concerns. Notably, despite being excluded from our analysis, several studies revealed promising findings regarding nonselective pelvic angioembolization. These studies demonstrated a high rate of hemostasis and a low rate of ischemic complications, particularly when using gelfoam material, compared to coils.

Nonselective embolization could be a reasonable choice for embolization in damage control scenarios to manage patients with severe injuries, owing to its expeditious and effective nature [7]. Recently, Wu et al. reported that pelvic injury prognosis is more closely related to vascular injury than anatomical fracture complexity [25]. Thus, expeditious bleeding control is crucial. Selective angiographic embolization can be time-consuming in a patient group requiring rapid hemostasis, and its success can be heavily dependent on the operator’s experience and proficiency in accurately discerning and accessing the anatomical structures. In contrast, nonselective embolization facilitates efficacious hemorrhage control in a reduced time span by inducing occlusion in the proximal part of the patient [7]. In addition, this nonselective embolization therapy can be applied not only to unilateral cases but also to the embolization of the bilateral internal iliac arteries when using agents that temporarily occlude the larger branches of the internal iliac artery. These temporarily occluding agents leave collateral blood flow and prevent significant ischemia with subsequent recanalization. In the most recent meta-analysis, PPP and embolization did not differ in terms of hemorrhage-related mortality [26]. Proximal embolization could serve as a faster alternative for patients in need of surgeries such as PPP.

However, nonselective angioembolization does not always yield favorable results. Our meta-analysis showed that nonselective angioembolization was associated with higher in-hospital mortality. Substantial selection bias and confounding factors may exist, but the eligible studies did not report an explicit indication for nonselective angioembolization. Nonselective angioembolization may be performed for more severe pelvic hemorrhages. Fang et al. [27] reported that patients who underwent repeat TAE were more likely to have a high mortality rate (15% in single TAE vs. 35% in repeated TAE, *p* = 0.02). They demonstrated that initial super-selective TAE was an independent predictor of repeated TAE in a multiple logistic regression model (OR 3.22, *p* = 0.005). In contrast, Fu et al. observed that nonselective and temporary embolization may elevate the risk of requiring repeated angioembolization [21]. The presence of combined injuries merits further consideration. Most patients with pelvic injuries require additional surgery and fixation beyond controlling bleeding through embolization. Nonselective angioembolization demonstrates a comparable risk of major pelvic ischemic complications to selective angioembolization. However, accurately predicting the risks associated with concurrent pelvic or hip surgery remains a challenging task. A study conducted by Lindvall et al. reported a high complication rate when nonselective internal iliac artery embolization was combined with pelvic and/or acetabular surgery [17]. Maruhashi et al. [24] demonstrated that the times required for TAE and external pelvic fixation surgery were independent risk factors for gluteal necrosis in a multiple logistic regression model (OR 1.030, *p* = 0.036; OR 8.374, *p* = 0.005, respectively). We noted that Maruhashi et al. reported no gluteal necrosis and they used a 2-mm gelatin sponge instead of a metallic coil as an embolic agent [24]. In contrast, Takahira et al. [19] reported that gluteal necrosis occurred at the site of embolization using a steel coil. Lindvall et al. [17] reported 10 patients with pelvic complications who underwent embolization using gelfoam.

In several studies [18,21,22,24] that were systematically reviewed but not included in the final meta-analysis, gelfoam was used during nonselective embolization, with results indicating minimal complications. Gelfoam is believed to temporarily block the major branches of the internal iliac artery [7]. This strategy allows smaller branches to remain open to collateral blood flow, with the aim of preventing severe ischemia. The transitory nature of absorbable gelatin, which constitutes gelfoam, permits occluded vessels to recanalize after several days or weeks [7]. This feature is particularly suitable for trauma patients to temporarily control bleeding rather than permanently occlude blood flow. However, the transient nature of absorbable gelatin may be a limitation in certain clinical settings. In the presence of severe trauma-induced coagulopathy, the temporary occlusion provided by gelfoam may not be sufficient to effectively control bleeding. This underlines the need for careful patient selection and further investigation into the optimal use of gelfoam for pelvic angioembolization.

In addition to gelfoam and coils, the recent advancements in new embolic agents might enhance the potential of pelvic embolization [28,29,30]. Liquid embolic agents such as Onyx, Squid, or PHIL have been introduced as effective treatments for cerebral arteriovenous malformation and arteriovenous fistulae. However, these are not widely used for pelvic embolization. In our review, there was no study that utilized liquid embolic agents. Further studies are warranted.

The existing literature suggests potential variations in outcomes based on the differences between unilateral and bilateral procedures [31]. However, our systematic analysis did not reveal significant differences between the unilateral and bilateral approaches owing to the limited sample sizes of the eligible studies. Therefore, further research on this topic is required.

Our study had several limitations. First, all studies included in our analysis were observational in nature, and no randomized controlled trials were incorporated. However, conducting randomized controlled trials in patients with severe pelvic injury accompanied by hemorrhage poses significant challenges in clinical settings. Second, it is important to note that certain studies included in our analysis had a moderate to serious risk of bias. To address this issue, we performed a sensitivity analysis. Nonetheless, the limited number of studies with a low risk of bias may restrict the generalizability of our findings. Third, our study was limited by the absence of data on the time from injury to embolization. The period between injury and intervention could potentially influence outcomes, as the rapid initiation of angioembolization may improve survival rates and reduce complications [32]. However, owing to the limited data in the included studies, we could not perform a detailed assessment of this potentially crucial factor. Fourth, the analysis of publication bias was constrained by the relatively small number of eligible studies, which may have introduced statistical instability and affected the interpretation of the results. Finally, we exclusively included articles published in English, which may have introduced language bias.

## 5. Conclusions

Despite the favorable outcomes observed with nonselective angioembolization for embolization-related pelvic complications, our systematic review and meta-analysis had substantial limitations in determining the effect sizes. These limitations stemmed from a significant risk of bias and heterogeneity among the included studies. Despite these challenges, encouraging findings emerged, such as the low occurrence of ischemic pelvic complications and high rate of successful hemostasis achieved through nonselective angioembolization in selective cases. Nonselective angioembolization could offer faster control of bleeding, with ischemic complications comparable to selective angioembolization. However, existing evidence necessitates further prospective investigations to effectively address these limitations. Future studies should minimize bias and heterogeneity. Nonetheless, we anticipate that our study findings will offer valuable insights to guide clinical decision making and optimize care for patients with pelvic injury and accompanying hemorrhage.

## Figures and Tables

**Table 1:** Main characteristics of the eligible studies.

Author	Year	Location	Study Period	Study Design	Inclusion and Exclusion	Hemodynamic Status	Injury Severity	Modality (Embolic Agent)	Indication of Nonselective Embolization	Primary and Secondary Outcomes
Travis [13]	2008	USA	1994–2006	obs, single center, comparative	Inclusion: Patients who underwent pelvic angiography after blunt trauma Exclusion: NR	NR	ISS: NR Pelvic AIS: NR	Nonselective (BIIA or UIIA) (33 patients) vs. selective embolization (21 patients) (gelfoam)	NR	Short-term outcome (<30 day)—pelvic or perineal infeciton, nerve damage, skin necrosis; Long-term outcome (>30 days)—pain, paresthesia, ulceration
Auerbach [14]	2012	USA	2004–2009	obs, single center, comparative	Inclusion: Patients who underwent TAE after blunt trauma Exclusion: Death within 48 h, no evidence of pelvic fracture	NR	ISS: Nonselective (13–41) vs. selective (9–29) Pelvic AIS: NR	Nonselective (UIIA) (2 patients) vs. selective embolization (18 patients) (coil (14 procedures) or gelfoam)	NR	Ischemic complications (gluteal necrosis, infection)
Shi [15]	2016	USA	2003–2013	obs, single center, comparative	Inclusion: Patients who underwent pelvic angiography Exclusion: <18 years old, death within 48 h, patients transferred from external hospitals, those who underwent angiography without embolization, those who had indications other than pelvic trauma, those who died from immediate complications related to their trauma (e.g., traumatic brain injury)	Lowest SBP: Nonselective, 87.9 (±21.1) vs. selective, 90.2 (±28.4); *p* < 0.20	ISS: Nonselective, 30.2 (±11.0) vs. selective, 22.7 (±7.4); *p* < 0.01 Pelvic AIS: NR	Nonselective (BIIA or UIIA) (55 patients) vs. selective embolization (33 patients) (gelfoam (70.1%] or coil)	NR	Major pelvic complications (gluteal necrosis, bladder necrosis, rectal necrosis, skin necrosis, surgical wound breakdown, superficial wound infection, deep soft tissue infection, abscess formation) during hospitalization or within 1 year
Hymel [16]	2017	USA	2002–2014	obs, multicenter (3 level 1 trauma centers), comparative	Inclusion: Blunt trauma with pelvic fracture undergoing angiography Exclusion: <18 years old	Initial SBP: Nonselective, 120.7 (±27.7) vs. selective, 118.9 (±29.7); *p* = 0.727	ISS: Nonselective, 25.9 (±11.2) vs. selective, 27.4 (±13.9); *p* = 0.483 Pelvic AIS: Nonselective, 2.8 (±0.9) vs. selective, 2.6 (±0.8); *p* = 0.128	Nonselective (BIIA or UIIA) (99 patients) vs. selective embolization (46 patients) (no information about embolic agent)	NR	Mortality; hemorrhagic control; embolization-related complications (short-term: wound infection or breakdown, gluteal or skin nerosis, and osteomyelitis; long-term: claudicationm sexual dysfucntion, paresthesia, pain, urinary dysfuction, wound infection or breakdown, fracture nonunion, or ostermyelitis); thromboembolic complications
Lindval [17]	2018	USA	2007–2014	obs, single center, comparative	Inclusion: Patients presenting with trauma activation with an associated pelvic and/or acetabular fracture who underwent pelvic angiography. All patients underwent open reduction internal fixation of their pelvic/acetabular fractures Exclusion: Death during initial hospital stay, loss to follow-up	NR	ISS: NR Pelvic AIS: NR	Nonselective (BIIA) (48 patients) vs. selective (2 patients) (gelfoam or coil)	NR	Surgical complications in patients undergoing surgical fixation of a pelvic and/or acetabular fracture after TAE

obs, observational; NR, not reported; TAE, transarterial angioembolization; SBP, systolic blood pressure; ISS, injury severity score; AIS, abbreviated injury scale; BIIA, bilateral internal iliac artery embolization; UIIA, unilateral internal iliac artery embolization.

**Table 2:** Excluded studies reporting nonselective pelvic embolization.

Author	Year	Location	Study Period	Study Design	Inclusion	Modality (Embolic Agent)	Indication of Nonselective Embolization	Complications	Mortality
Velmahos [18]	2000	USA	1991–1998	obs, single center	Inclusion: blunt pelvic trauma with BIIA Exclusion: patients with UIIA	Nonselective BIIA (30 patients) (all gelfoam)	1. Patients continued to require fluid and blood transfusions despite apparently successful sub-selective embolization of different branches 2. Mutiple bleeding sites bilaterally 3. Hemodynamic lability precluded technically challenging and time-consuming maneuvers to occlude small branches selectively	2 patients with hematoma at arterial access site, no severe complications, no ischmic complications, 2 patients with repeated embolization	10 patients (only one related uncontrolled bleeding)
Takahira [19]	2001	Japan	1979–1999	obs, single center	Inclusion: pelvic fracture with gluteal necrosis after BIIA	Nonselective BIIA (5) (gelfoam or coil)	Hemorrhagic shock	5 patients with gluteal necrosis	3 patients with gluteal necrosis (60%)
Suzuki [20]	2005	Japan	1995–2003	obs, single center	Inclusion: pelvic fracture managed with BIIA Exclusion: death within 48 h	Nonselective BIIA (132 patients) (all gelfoam)	Pelvic fracture with retroperitoneal bleeding	12 patients with gluteal muscle necrosis and skin necrosis	4/12 deaths among those with gluteal necrosis
Fu [21]	2013	Taiwan	2005–2011	obs, multi-center (2 hostpials)	Inclusion: patients with pelvic fracture undergoing computed tomography	Nonselective BIIA (27 patients), selective UIIA (43 patients) (gelfoam or coil)	Bilateral contast extravasation on CT scan	Repeated TAE: 1/27 in nonselective BIIA, 7/43 in selective UIIA, no long-term complications in nonselective BIIA, 1 skin ulcer in BIIA	NR
Bonde [22]	2020	USA	1998–2018	obs, single center	Inclusion: pelvic fracture managed with BIIA Exclusion: angiography without embolization, unilateral embolization	Nonselective BIIA (61 patients) (gelfoam [all] or coil [additional])	Pelvic fracture with significant bleeding and labile hemodynamics	10 patients with ongoing bleeding, 4 patiens with re-angiography, no pelvic/gluteal/perineal necrosis	6 patients died with pelvic bleeding, a total of 18 patients died (30%)
Lai [23]	2020	Taiwan	2014–2017	obs, single center	Inclusion: pelvic fracture managed with AE Exclusion: dead on arrival, isolated acetabular fracture, diagnosed with pelvic fracture without imaging, AE as a hemostatic procedure targeting non-pelvic regions	Nonselective BIIA (97 patients) (gelfoam [generally] or coil)	Discretion of the inverventional radiologist	9/11 SSIs related to BIIA	18/129 (13.7%) among patients with AE
Maruhashi [24]	2020	Japan	2005–2015	obs, single center	Inclusion: pelvic fracture managed with BIIA Exclusion: selective embolization, embolic agent other than gelatin sponge	Nonselective BIIA (70 patients) (only gelfoam)	Hemodynamic instability or extravasation on CT	No gluteal necrosis	Overall 12/70 (17.1%)

obs, observational; BIIA, bilateral internal iliac artery embolization; AE, angioembolization; UIIA, unilateral internal iliac artery embolization; TAE, transarterial angioembolization; NR, not reported.

## Data Availability

Not applicable.
